# Diet quality is positively associated with 100% fruit juice consumption in children and adults in the United States: NHANES 2003-2006

**DOI:** 10.1186/1475-2891-10-17

**Published:** 2011-02-13

**Authors:** Carol E O'Neil, Theresa A Nicklas, Michael Zanovec, Victor L Fulgoni

**Affiliations:** 1 School of Human Ecology, Louisiana State University Agricultural Center, 261 Knapp Hall, Baton Rouge, LA 70803, USA; 2 USDA/ARS Children's Nutrition Research Center, Department of Pediatrics, Baylor College of Medicine, 1100 Bates Avenue, Houston, TX 77030, USA; 3 Nutrition Impact, LLC, 9725 D Drive North, Battle Creek, MI 49014, USA

## Abstract

**Background:**

One hundred percent fruit juice (100% FJ) has been viewed by some as a sweetened beverage with concerns about its effect on weight. Little regard has been given to the contribution of 100% FJ to diet quality.

**Methods:**

In this study data from the 2003-2006 National Health and Nutrition Examination Survey were used to examine the association of 100% FJ consumption with diet quality in participants 2-5 years of age (y) (n = 1665), 6-12 y (n = 2446), 13-18 y (n = 3139), and 19+y (n = 8861). Two 24-hour dietary recalls were used to determine usual intake using the National Cancer Institute method. Usual intake, standard errors, and regression analyses (juice independent variable and Healthy Eating Index-2005 [HEI-2005] components were dependent variables), using appropriate covariates, were determined using sample weights.

**Results:**

The percentage of participants 2-5 y, 6-12 y, 13-18 y, and 19+y that consumed 100% FJ was 71%, 57%, 45%, and 62%, respectively. Usual intake of 100% FJ (ounce [oz]/day) among the four age groups was: 5.8 ± 0.6, 2.6 ± 0.4, 3.7 ± 0.4, and 2.4 ± 0.2 for those in age groups 2-5 y, 6-12 y, 13-18 y, and 19+y, respectively. Consumption of 100% FJ was associated with higher energy intake in 6-12 y, 13-18 y, and 19+y; and higher total, saturated, and discretionary fats in 13-18 y participants. Consumption of 100% FJ was associated with higher total HEI-2005 scores in all age groups (< 0.0001). In 100% FJ consumers, total and whole fruit consumption was higher and intake of added sugars was lower in all age groups.

**Conclusions:**

Usual intake of 100% FJ consumption exceeded MyPyramid recommendations for children 2-5 y, but was associated with better diet quality in all age groups and should be encouraged in moderation as part of a healthy diet.

## Background

Consumption of fruit is associated with a variety of health benefits including an improved profile of cardiovascular disease markers [1], and a reduced risk of hypertension [2] and some types of cancer [3]. Despite these health benefits, few Americans consume the recommended amounts of fruit per day [4-6]. The MyPyramid recommendations for fruit consumption are age, gender, and physical activity specific [7]. Recommendations range from 1 cup/day for children 2-3 years of age (y) to a maximum of 2.5 cups/day for physically active young males. The fruit requirement can be met by consuming whole fruit--fresh, frozen, or dried, or 100% fruit juice (FJ); although the Dietary Guidelines Advisory Committee (DGAC) recommended that only 1/3 of the recommendation should be met through 100% FJ to encourage fiber intake [8]. The American Academy of Pediatrics (AAP) recommends that for children 1 to 6 y, 100% juice should be limited to 4 to 6 oz/day, and for older children/adolescents, 7 to 18 y, to two 6-oz servings of juice per day [9].

Recommendations for consumption of 100% FJ by children, however, continue to be debated due to concerns about consumption and a potential link with overweight/obesity. Although some studies have shown an association between 100% FJ consumption and weight [10-13], the majority have not [14-20]. A systematic review published in 2008 showed no consistent association between consumption of 100% FJ and overweight/obesity in children or adolescents [20]. Studies of weight and 100% FJ consumption have been primarily in children since they are the principal consumers. Few studies have looked at 100% FJ consumption and weight in adults [21-23], but there is less concern than in children.

What is often overlooked in the on-going debate about 100% FJ and weight and the uncertainty of how much, if any, should be consumed, is the nutrient contribution of 100% FJ to the diet. One hundred percent fruit juices are nutrient dense [24] and are low in total fat, SFA, and sodium. One hundred percent fruit juices, especially grape, cranberry, pineapple, and orange juices, are high in phytochemicals [25]. They also contain a wide array of micronutrients, including vitamins A (particularly in the form of beta-carotene) and C, folate, potassium, and magnesium. The 2010 DGAC recognized vitamins A, C, D, and E; phosphorus; magnesium; potassium; and dietary fiber [26] as shortfall nutrients, and in addition, potassium and dietary fiber were nutrients of public health concern. Calcium was also a nutrient of public health concern for children and adolescents 9 to 18 y, and possibly for younger children aged 4 to 8 y, as well as for adults [26]. For adolescents (and women) of childbearing potential, folate was also identified as a nutrient of special concern [26].

Previous studies of children 2-11 y [15] and adolescents 12-18 y [16] found that 100% FJ consumers had better nutrient intakes and higher intakes of whole fruit than those that did not consume 100% FJ. Although the association between 100% FJ intake and nutrient intake has been established in nationally representative samples, assessment and comparison of diet quality of 100% FJ consumers versus non-consumers is lacking. The purpose of this study was to compare the diet quality of 100% FJ consumers with non-consumers in a nationally representative population.

## Methods

### Data collection

The National Health and Nutrition Examination Survey (NHANES) is conducted on a continual basis by the National Center for Health Statistics of the Centers for Disease Control and Prevention. One of the major objectives of NHANES is to examine the relationship between diet, nutrition, and health [27]. Details regarding the survey design, content, operations and procedures are available online [28].

### Study population and dietary intake

Participants were 2 y and older (n = 16111) from the 2003-2006 NHANES. The study population was divided into four age groups and then further dichotomized as consumers and non-consumers of 100%FJ as follows: children 2-5 y (n = 1184 and 481, consumers and non-consumers, respectively); 6-12 y (n = 1395 and 1051); adolescents 13-18 y (n = 1397 and 1742); and adults19+ y (n = 3394 and 5467). Dietary data were obtained from two 24-hour dietary recalls administered using an automated multiple-pass method [29,30]. The first was obtained at the original interview (Day 1) and the second (Day 2) was obtained several days later via telephone. Parents/guardians provided the 24-hour dietary recalls of children 2-5 y; children 6-11 y were assisted by an adult; all others provided their own recalls. Only recall data deemed complete and reliable by the USDA Food Surveys Research Group were included in the analyses. Pregnant or lactating females (n = 711) were excluded from the sample. Detailed descriptions of the dietary interview methods are provided in the NHANES MEC In-Person Dietary Interviewers Procedures Manual, which includes pictures of the Computer-Assisted Dietary Interview system screens, measurement guides, and charts used to collect dietary information [31]. Due to the nature of the analysis (secondary data analysis), and the lack of personal identifiers, this study was exempted by the Institutional Review Board of the Louisiana State University Agricultural Center.

### Determination of 100% FJ and nutrient intake

Two survey-specific food composition databases were used to determine the foods consumed by NHANES participants. The USDA Food and Nutrient Database for Dietary Studies (FNDDS) v. 2.0 [32] was used to determine the nutrient content of foods in 2003-2004 NHANES survey foods, and the FNDDS v. 3.0 [33] was used to determine the nutrient content of foods contained in 2005-2006 NHANES survey foods.

In this study, 100% FJ was defined according to the Federal Food and Drug Administration [34], which means that the juice was squeezed directly from fruit and that the words "100% juice" were included on the label. Products reconstituted from concentrate with water were also considered 100% FJ, although their label must have included the words "reconstituted" or "made from concentrate." Juice cocktails, juice punches, juice drinks, or juice beverages were not considered as fruit juice in this study, although they contain some juice [34]. Participants were dichotomized into consumers and non-consumers of 100% FJ. Further, the amount of 100% FJ consumed was compared with the MyPyramid recommendation that 100% FJ should not account for more than 1/3 of total fruit intake per day, which is equivalent to the following amounts in cups (c) per total daily energy levels: 0.33 c/1000-1200 kcal; 0.495 c/1400-1800 kcal; 0.66 c/2000-2600 kcal; and 0.825 c/2800-3200 kcal [7].

### Healthy eating index

The Healthy Eating Index (HEI-2005) was used to determine diet quality [35]. The HEI-2005 contains 12 food components that reflect the recommendations of the 2005 Dietary Guidelines for Americans. Dietary intake is expressed per 1000 kilocalories for all components except SFA and sodium, which are fixed recommendations. The maximum possible score on the index is 100. The first six components (*i.e.*, total fruit; whole fruit; total vegetable; dark green, orange vegetable and legumes; total grain; and whole grain) were scored from 0 to 5 points. The next five components (*i.e.*, milk, meat and beans, oil, SFA, and sodium) were scored from 0 to 10 points; and the last component of solid fats, alcoholic beverages, and added sugars (SoFAAS) were scored from 0 to 20 points. Scores were calculated proportionally, except for SFA and sodium; for these components, scores were prorated linearly between 0 to 8 and 8 to 10 points (8 and 10 points represented acceptable and optimal levels, respectively) [36]. Data files used to calculate HEI-2005 scores were downloaded from the USDA Center for Nutrition Policy and Promotion website [37].

### Statistical analyses

The National Cancer Institute (NCI) method was used to estimate usual intake of selected nutrients and for HEI-2005 total scores and subcomponent scores. The two days of intake, using first day sampling weights, were used to obtain necessary variance estimates. The NCI SAS (SAS Institute, Inc., Cary, NC) macros Mixtran v.1.1 and Distrib v.1.1 were used to generate parameter estimates after covariate adjustment and to estimate the distribution of usual intake via the Monte Carlo method, respectively. Covariates for these analyses were sequence of participant's intake (Day 1 or Day 2) and a variable for weekday/weekend consumption. Differences among 100% FJ consumers and non-consumers were determined by computing population Z statistics generated from usual intake variables.

Regression analyses were conducted using the single 24-hour recall data (Day 1) to determine the contribution of nutrients per ounce of 100% FJ consumption. Covariates for the regression analyses were age, gender, race-ethnicity, and socioeconomic status (using poverty income ratio). Differences in beta coefficients were determined and p < 0.05 was deemed significant. For all analyses study-specific dietary four-year sample weights [38] were used to adjust the variance for the complex sample design of NHANES using the statistical package SUDAAN (version 9.0.3 [2007] Research Triangle Institute, Research Triangle Park, NC).

## Results

### Consumption of 100% fruit juice

The percentage of individuals consuming 100% FJ varied by age group, with 71%, 57%, 45%, and 62% of children 2-5 y, 6-12 y, 13-18 y, and adults 19+ y, respectively. Per capita usual intake of 100% FJ (oz) among the four age groups was: 5.8 ± 0.6, 2.6 ± 0.4, 3.7 ± 0.4, and 2.4 ± 0.2 for those 2-5 y, 6-12 y, 13-18 y, and 19+ y, respectively. Usual intake of 100% FJ (oz) among only those consuming 100% FJ was higher than per capita intake: 8.5 ± 0.8, 5.4 ± 0.8, 10.0 ± 1.0, and 7.1 ± 0.5 for those 2-5 y, 6-12 y, 13-18 y, and 19+ y, respectively (data not shown).

### Intake of energy, fiber, added sugars, and fat

Usual energy intake (kcal) of 100% FJ consumers was 1657.6 ± 47.2, 2079.5 ± 52.8, 2718.0 ± 77.5, and 2348.7 ± 47.1; for non-consumers energy intake was 1627.5 ± 72.8, 2089.0 ± 66.9, 2413.5 ± 65.1, 2297.0 ± 29.7 for age groups 2-5 y, 6-12 y, 13-18 y, and 19+ y, respectively (Table 1). Fiber intake (g) of 100% FJ consumers was 10.3 ± 0.4, 12.9 ± 0.4, 15.9 ± 0.8, and 16.5 ± 0.4; for non-consumers intake was 10.2 ± 0.7, 12.9 ± 0.6, 13.2 ± 0.6, and 15.1 ± 0.4 for age groups 2-5 y, 6-12 y, 13-18 y, and 19+ y, respectively. Usual intake of added sugars (tsp) by 100% FJ consumers was 15.3 ± 0.6, 24.1 ± 1.1, 29.5 ± 1.3, and 20.4 ± 0.8; for non-consumers added sugars intake was 18.7 ± 1.2, 27.2 ± 1.8, 32.3 ± 1.3, 23.1 ± 0.6 for age groups 2-5 y, 6-12 y, 13-18 y, and 19+ y, respectively. Usual intake of total fat (g) by 100% FJ consumers was 56.5 ± 2.2, 75.9 ± 3.2, 103.3 ± 3.7, and 87.9 ± 1.9; for non-consumers total fat intake was 63.5 ± 3.7, 78.8 ± 2.8, 91.8 ± 2.8, 88.5 ± 1.9 for age groups 2-5 y, 6-12 y, 13-18 y, and 19+ y, respectively. Usual intake of SFA (g) by 100% FJ consumers was 20.9 ± 0.9, 26.9 ± 1.2, 35.9 ± 1.4, and 28.9 ± 0.7; for non-consumers SFA intake was 23.6 ± 1.6, 27.9 ± 1.0, 31.4 ± 0.9, 29.3 ± 0.7 for age groups 2-5 y, 6-12 y, 13-18 y, and 19+ y, respectively. Usual intake of discretionary fat (g) by 100% FJ consumers was 48.0 ± 2.0, 64.4 ± 2.9, 86.3 ± 3.2, 69.9 ± 1.5; for non-consumers discretionary fat intake was 54.9 ± 3.9, 68.0 ± 2.6, 76.7 ± 2.2, 71.2 ± 1.6 for age groups 2-5 y, 6-12 y, 13-18 y, and 19+ y, respectively (Table 1).

**Table 1:** Distribution of usual intake (UI) for total energy and select nutrients among 100% FJ consumers and non-consumers by age group: NHANES 2003-2006

					Percentiles				
Consumers/Non-consumers	Age group (y)	n	UI	SE	10	25	50	75	90
**Energy (kcal)**									
**Consumers**	2-5 y	1184	1657.6	47.2	1225.8	1407.3	1629.5	1880.0	2124.3
	6-12 y	1395	2079.5	52.8	1582.1	1797.8	2054.1	2333.5	2606.1
	13-18 y	1397	2718.0	77.5	1740.5	2121.7	2616.1	3200.0	3825.1
	19+ y	3394	2348.7	47.1	1488.8	1826.5	2266.8	2780.6	3314.4
**Non-consumers**	2-5 y	481	1627.5	72.8	1159.7	1353.0	1594.6	1865.8	2137.6
	6-12 y	1051	2089.0	66.9	1585.1	1807.6	2069.6	2349.4	2613.6
	13-18 y	1742	2413.5	65.1	1516.3	1877.9	2337.5	2871.1	3402.8
	19+ y	5467	2297.0	29.7	1411.4	1761.1	2214.9	2742.8	3288.5
**Fiber (g)**									
**Consumers**	2-5 y	1184	10.3	0.4	6.6	8.1	10.0	12.2	14.4
	6-12 y	1395	12.9	0.4	8.9	10.5	12.6	15.0	17.3
	13-18 y	1397	15.9	0.8	9.4	11.8	15.1	19.1	23.5
	19+ y	3394	16.5	0.4	9.4	12.1	15.7	20.0	24.5
**Non-consumers**	2-5 y	481	10.2	0.7	6.2	7.8	9.8	12.1	14.7
	6-12 y	1051	12.9	0.6	9.6	11.0	12.7	14.7	16.6
	13-18 y	1742	13.2	0.6	7.6	9.8	12.7	16.1	19.6
	19+ y	5467	15.1	0.4	8.1	10.8	14.3	18.6	23.0
**Added sugars (tsp)**									
**Consumers**	2-5 y	1184	15.3	0.6	8.4	11.0	14.5	18.7	23.0
	6-12 y	1395	24.1	1.1	14.4	18.3	23.3	29.0	34.9
	13-18 y	1397	29.5	1.3	15.4	20.7	28.0	36.8	45.8
	19+ y	3394	20.4	0.8	7.4	11.6	18.1	26.6	36.4
**Non-consumers**	2-5 y	481	18.7	1.2	8.2	12.0	17.4	23.9	30.8
	6-12 y	1051	27.2	1.8	16.4	20.7	26.3	32.7	39.3
	13-18 y	1742	32.3	1.3	15.3	21.7	30.4	40.7	51.7
	19+ y	5467	23.1	0.6	7.2	12.2	19.9	30.6	43.0
**Total fat (g)**									
**Consumers**	2-5 y	1184	56.5	2.2	37.3	45.0	55.0	66.3	77.8
	6-12 y	1395	75.9	3.2	54.3	63.5	74.8	87.0	98.8
	13-18 y	1397	103.3	3.7	61.7	77.8	99.1	124.4	150.5
	19+ y	3394	87.9	1.9	51.2	65.4	84.2	106.3	129.5
**Non-consumers**	2-5 y	481	63.5	3.7	42.7	51.2	61.9	74.2	86.5
	6-12 y	1051	78.8	2.8	55.6	65.6	77.7	90.7	103.5
	13-18 y	1742	91.8	2.8	56.2	70.5	88.8	109.7	131.1
	19+ y	5467	88.5	1.9	50.1	65.1	84.7	107.8	131.7
**SFA (g)**									
**Consumers**	2-5 y	1184	20.9	0.9	13.1	16.2	20.2	24.8	29.5
	6-12 y	1395	26.9	1.2	18.9	22.3	26.5	31.0	35.5
	13-18 y	1397	35.9	1.4	21.0	26.8	34.5	43.4	52.6
	19+ y	3394	28.9	0.7	15.9	20.8	27.4	35.4	43.9
**Non-consumers**	2-5 y	481	23.6	1.6	15.5	18.8	23.0	27.7	32.6
	6-12 y	1051	27.9	1.0	19.4	23.0	27.5	32.3	37.1
	13-18 y	1742	31.4	0.9	18.8	23.8	30.4	37.9	45.5
	19+ y	5467	29.3	0.7	15.8	20.9	27.8	36.1	44.9
**Discretionary fat (g)**									
**Consumers**	2-5 y	1184	48.0	2.0	31.1	38.0	46.7	56.5	66.5
	6-12 y	1395	64.4	2.9	44.6	53.0	63.2	74.6	85.6
	13-18 y	1397	86.3	3.2	50.1	64.1	82.6	104.6	127.2
	19+ y	3394	69.9	1.5	38.6	50.7	66.7	85.6	105.3
**Non-consumers**	2-5 y	481	54.9	3.9	36.6	44.2	53.7	64.2	75.0
	6-12 y	1051	68.0	2.6	47.0	56.0	66.9	78.6	90.3
	13-18 y	1742	76.7	2.2	46.0	58.3	74.1	92.4	110.7
	19+ y	5467	71.2	1.6	39.2	51.6	68.1	87.4	107.4

Data are presented as sample-weighted mean usual intake ± SE, and percentiles of usual intake. Abbreviations: kcal, kilocalories; tsp, teaspoon; g, grams; NHANES, National Health and Nutrition Examination Survey; y, years; n, number; UI, usual intake; SE, standard error; SFA, saturated fatty acids. *Note*: There are 4.2 g in one tsp of sugar.

In all age groups, except those 2-5 y, consumption of 100% FJ was associated with significantly higher intakes of energy: in 6-12 y (80.9 ± 31.6 kcal; p = 0.0105), in 13-18 y (252.3 ± 49.9 kcal; p < 0.0001), and in adults 19+ y (120.9 ± 23.7 kcal; p < 0.0001) (Table 2). Except in the youngest age groups, consumption of 100% FJ was associated with higher intake of dietary fiber: 1.3 ± 0.3 g (p < 0.0001), 2.5 ± 0.4 g (p < 0.0001), and 1.8 ± 1.4 g (p < 0.0001), for the three other age groups, respectively. One hundred percent FJ consumption was also associated with lower intake of added sugars (tsp) in all age groups: -4.6 ± 0.7 (p < 0.0001) in 2-5 y, -2.8 ± 0.7 (p < 0.0001) in 6-12 y, -1.8 ± 0.9 (p = 0.0396) in 13-18 y and -1.0 ± 0.4 (p = 0.0154) in 19+ y, respectively.

**Table 2:** Regression analysis of 100% FJ consumption (any amount) with total energy and select nutrients by age group: NHANES 2003-2006

	Juice consumers (> 0 oz/day)											
	2-5 y			6-12 y			13-18 y			19+ y		
	(n = 1184)			(n = 1395)			(n = 1397)			(n = 3394)		
Variable	β	SE	*P*-value	β	SE	*P*-value	β	SE	*P*-value	β	SE	*P*-value
Energy (kcal)	39.6	35.9	0.2709	80.9	31.6	0.0105	252.3	49.9	< 0.0001	120.9	23.7	< 0.0001
Fiber (g)	0.7	0.4	0.06	1.3	0.3	< 0.0001	2.5	0.4	< 0.0001	1.8	1.4	< 0.0001
Added sugars (tsp)	-4.6	0.7	< 0.0001	-2.8	0.7	< 0.0001	-1.8	0.9	0.0396	-1.0	0.4	0.0154
Total fat (g)	-1.5	1.7	0.4026	0.9	1.5	0.5430	6.3	2.2	0.0035	0.8	1.1	0.4580
SFA (g)	-0.8	0.7	0.2371	0.5	0.6	0.3980	1.9	0.8	0.0152	-0.15	0.39	0.7034
Discretionary fat (g)	-1.7	1.5	0.2367	-0.4	1.4	0.7890	4.8	1.9	0.0118	0.1	0.9	0.9417

Data are presented as unstandardized regression coefficients (β), standard errors (SE), and *P*-values of β coefficients. Abbreviations: FJ, fruit juice; y, years; SE, standard error; kcal, kilocalories; tsp, teaspoon; g, gram; SFA, saturated fatty acids. *Note*: There are 4.2 g in one tsp of sugar.

Adolescents 13-18 y were the only age group in which consumers of 100% FJ had a higher intake of total fat, SFA, and discretionary fat than non-consumers (Table 1).

### Diet quality

The HEI-2005 scores of 100% FJ consumers by age were 53.0 ± 1.2, 49.3 ± 1.7, 49.6 ± 0.9, and 52.6 ± 0.6, for age groups 2-5 y, 6-12 y, 13-18 y, and 19+ y, respectively (Table 3). The HEI-2005 scores of non-consumers by age were 47.3 ± 2.1, 44.1 ± 1.7, 44.4 ± 1.0, and 47.7 ± 0.5, for age groups 2-5 y, 6-12 y, 13-18 y, and 19+ y, respectively. For all age groups, consumers of 100% FJ had higher HEI-2005 scores than non-consumers, even when HEI-2005 scores were compared across percentiles (data not shown).

**Table 3:** Comparison of HEI-2005 total and select subcomponent usual intake (UI) scores between 100% FJ consumers and non-consumers by age group: NHANES 2003-2006

		Consumers			Non-Consumers		
Variable	Age group (y)	n	UI score	SE	n	UI score	SE
HEI-2005--Total Score	2-5 y	1184	53.0	1.2	481	47.3	2.1
	6-12 y	1395	49.3	1.7	1051	44.1	1.7
	13-18 y	1397	49.6	0.9	1742	44.4	1.0
	19+ y	3394	52.6	0.6	5467	47.7	0.5
HEI-2005--Total Fruit	2-5 y	1184	4.1	0.1	481	1.8	0.2
	6-12 y	1395	3.0	0.3	1051	1.4	0.2
	13-18 y	1397	3.2	0.3	1742	1.1	0.1
	19+ y	3394	3.0	0.2	5467	1.3	0.1
HEI-2005--Whole Fruit	2-5 y	1184	2.4	0.2	481	1.8	0.3
	6-12 y	1395	1.9	0.2	1051	1.8	0.3
	13-18 y	1397	1.7	0.7	1742	1.2	0.1
	19+ y	3394	2.2	0.1	5467	1.6	0.1
HEI-2005--Milk	2-5 y	1184	7.8	0.2	481	8.2	0.2
	6-12 y	1395	7.0	0.3	1051	6.4	0.3
	13-18 y	1397	5.9	0.3	1742	5.9	0.3
	19+ y	3394	4.9	0.2	5467	4.6	0.2
HEI-2005--SFA	2-5 y	1184	5.7	0.6	481	4.0	0.8
	6-12 y	1395	5.0	0.4	1051	5.2	0.5
	13-18 y	1397	5.3	0.3	1742	5.4	0.2
	19+ y	3394	6.0	0.2	5467	5.9	0.2
HEI-2005--Sodium	2-5 y	1184	5.1	0.3	481	4.6	0.4
	6-12 y	1395	4.4	0.3	1051	4.6	0.4
	13-18 y	1397	4.6	0.3	1742	4.8	0.3
	19+ y	3394	4.6	0.2	5467	4.1	0.1
HEI-2005--SoFAAS	2-5 y	1184	10.1	0.6	481	6.8	0.9
	6-12 y	1395	7.7	0.6	1051	5.4	0.8
	13-18 y	1397	8.1	0.5	1742	5.6	0.6
	19+ y	3394	9.0	0.3	5467	7.6	0.3

Abbreviations: HEI-2005, Healthy Eating Index-2005; y, years; n, number; UI, usual intake; SE, standard error; SFA, saturated fatty acids; SoFAAS, solid fats, alcoholic beverages, and added sugars.

Results of the regression analysis of 100% FJ consumption with total HEI-2005 and HEI-2005 subcomponent scores by age group are presented in Table 4. Consumption of 100% FJ contributed to the total HEI-2005 score in each age group by 7.1 ± 0.8, 5.5 ± 0.5, 5.6 ± 0.5, and 5.1 ± 0.3 points, respectively (p < 0.0001 for all). Total fruit scores were higher in each age group by 1.9 ± 0.1, 1.7 ± 0.1, 2.0 ± 0.1, and 1.7 ± 0.1, respectively (p < 0.001 for all). Whole fruit scores were also higher in all ages groups by 0.5 ± 0.1 (p = 0.0006), 0.3 ± 0.1 (p = 0.0010), 0.3 ± 0.1 (p = 0.0004), and 0.4 ± 0.05 (p < 0.0001), respectively. The dark green/orange vegetables/legumes scores were higher in children 6-12 y (0.2 ± 0.1 [p = 0.0007]) and adults 19+ y (0.1 ± 0.1 [p = 0.0030]), but not in the other age groups (data not shown). Whole grains scores were significantly higher in children 2-5 y (0.2 ± 0.1 [p = 0.0323]), 6-12 y (0.2 ± 0.1 [p = 0.0011]), and adults 19+ y (0.2 ± 0.1 [p < 0.0001]) (data not shown). The most significant contribution to the overall HEI-2005 score was the improvement in the SoFAAS score: 3.4 ± 0.4, 2.3 ± 0.3, 2.3 ± 0.3, and 1.7 ± 0.2, respectively in the four age groups (p < 0.0001 for all).

**Table 4:** Regression analysis of 100% FJ consumption with HEI-2005 total and subcomponent scores by age group: NHANES 2003-2006

	Juice consumers (> 0 oz/day)		
Variable	β	SE	*P*-value
**Total HEI-2005**			
2-5 y	7.1	0.8	< 0.0001
6-12 y	5.5	0.5	< 0.0001
13-18 y	5.6	0.5	< 0.0001
19+ y	5.1	0.3	< 0.0001
**Total fruit**			
2-5 y	1.9	0.1	< 0.0001
6-12 y	1.7	0.1	< 0.0001
13-18 y	2.0	0.1	< 0.0001
19+ y	1.7	0.1	< 0.0001
**Whole fruit**			
2-5 y	0.5	0.1	0.0006
6-12 y	0.3	0.1	0.0010
13-18 y	0.3	0.1	0.0004
19+ y	0.4	0.1	< 0.0001
**Milk**			
2-5 y	-0.2	0.2	0.3326
6-12 y	0.2	0.2	0.1881
13-18 y	-0.0	0.1	0.8766
19+ y	0.0	0.1	0.8867
**SFA**			
2-5 y	0.7	0.2	0.0019
6-12 y	0.3	0.2	0.0621
13-18 y	0.7	0.1	< 0.0001
19+ y	0.6	0.1	< 0.0001
**Sodium**			
2-5 y	0.4	0.2	0.0268
6-12 y	0.2	0.1	0.2173
13-18 y	0.5	0.1	0.0001
19+ y	0.4	0.1	< 0.0001
**SoFAAS**			
2-5 y	3.4	0.4	< 0.0001
6-12 y	2.3	0.3	< 0.0001
13-18 y	2.3	0.3	< 0.0001
19+ y	1.7	0.2	< 0.0001

Data are presented as unstandardized regression coefficients (β), standard errors (SE), and *P*-values of β coefficients.

Abbreviations: y, years; SE, standard error; HEI-2005, Healthy Eating Index-2005; SFA, saturated fatty acids; SoFAAS, solid fats, alcoholic beverages, and added sugars.

The distribution of total HEI-2005 usual intake scores of consumers and non-consumers of 100% FJ is shown in Table 5. In all age groups, consumers had a higher (p < 0.05) HEI-2005 score than non-consumers: 2-5 y (53.0 ± 1.2 vs. 47.3 ± 2.1), 6-12 y (49.3 ± 1.7 vs. 44.1 ± 1.7), 13-18 y (49.6 ± 0.9 vs. 44.4 ± 1.0), and 19+ y (52.6 ± 0.6 vs. 47.7 ± 0.5).

**Table 5:** Distribution of HEI-2005 total usual intake (UI) scores among 100% FJ consumers and non-consumers by age group: NHANES 2003-2006

		HEI-2005			Percentiles				
	Age group (y)	n	UI	SE	10	25	50	75	90
**Consumers**	2-5 y	1184	53.0 ^a^	1.2	44.0	48.3	53.0	57.7	61.9
	6-12 y	1395	49.3 ^a^	1.7	42.3	45.5	49.2	52.9	56.3
	13-18 y	1397	49.6 ^a^	0.9	41.2	45.0	49.5	53.9	58.0
	19+ y	3394	52.6 ^a^	0.6	41.5	46.7	52.5	58.5	63.8
**Non-consumers**	2-5 y	481	47.3 ^b^	2.1	38.8	42.5	47.0	51.7	56.4
	6-12 y	1051	44.1 ^b^	1.7	37.2	40.3	43.9	47.7	51.2
	13-18 y	1742	44.4 ^b^	1.0	36.6	40.1	44.1	48.5	52.5
	19+ y	5467	47.7 ^b^	0.5	36.4	41.5	47.4	53.5	59.4

^ab^Usual HEI-2005 means with different superscripts within age groups are significantly (p < 0.05) different. Abbreviations: y, years; n, number; UI, usual intake; SE, standard error.

## Discussion

This study showed that the percentage of 100% FJ consumers was age dependent; children 2-5 y had the highest percentage of consumers and adolescents 13-18 y had the lowest percentage of consumers. Diet quality, determined by the HEI-2005, was better in all age groups of 100% FJ consumers when compared with non-consumers.

The age variation of the percentage of 100% FJ consumers is consistent with other studies [15,16]. Reasons for the high prevalence of 100% FJ consumption in children may include participation in the Women, Infants, and Children (WIC) Program by young children [39] since 100% FJ is a WIC authorized food in three of the food packages for children over 11 months of age [40]. Participation in the National School Lunch or Breakfast Program by older children [41] may also lead to consumption of 100% FJ since these are approved menu items, and is the only juice product that may be served in the National School Breakfast Program [42]. It is also possible the frequency of consumption is lowest in adolescents since they are the least likely age group to consume breakfast [43] and 100% FJ is thought of as a breakfast food by many consumers. Adolescents who participate in the National School Lunch program also have more beverage options than elementary school children [44] have and may not choose 100% FJ. Studies have also shown a secular decline in consumption of 100% FJ by adolescents over the past five years [45].

MyPyramid [7] and AAP [9] recommendations state that 100% FJ can be part of a healthy diet when served in age appropriate amounts. This study showed that the usual intake for children 2-5 y exceeded these recommendations. Further studies are needed to assess the health effects of consuming higher than recommended levels of 100% FJ, and whether consuming whole fruit without 100% FJ provides an advantage to consumers. Recommendations for 100% FJ consumption vary; thus, caretakers and consumers may be confused and efforts should be made to reconcile the recommendations using an evidence-based approach.

MyPyramid recommendations were established, in part, to encourage fiber intake since modeling studies by the USDA showed that when 100% FJ was replaced by whole fruit, fiber intake increased by nearly 37% [8]. Regression analysis did show a modest, but significant contribution of fiber to the diet from 100% FJ in all but the youngest age group. That there was no difference in fiber intake between 100% FJ consumers and non-consumers suggests that other foods were contributing fiber to the diet. Usual fiber intake was, however, below the Institute of Medicine's recommendations [46] in all age groups in 100% FJ consumers and non-consumers and foods rich in fiber should be encouraged.

The rationale for the AAP recommendations for 100% FJ consumption in children and adolescents is not completely clear, but is in part based on one study that showed an association of 100% FJ consumption and overweight in pre-school aged children [10]. Although concerns about overweight/obesity and consumption of 100% FJ appear unfounded [20], it is important to consume 100% FJ with other foods while maintaining energy balance. Paradoxically, in all age groups except children 2-5 y, the group that consumed the most 100% FJ, energy was higher in 100% FJ consumers. The relationship of weight and 100% FJ consumption was not examined in this study.

Previous studies have shown that children [15] and adolescents [16] also had higher intakes of total fruit and adolescents had higher intakes of citrus, melons, and berries. These studies have not been conducted in adults. Moreover, studies have not examined the effect of 100% FJ intake on overall diet quality. The HEI-2005, used to assess diet quality, was revised to reflect the 2005 Dietary Guidelines [47]. The HEI-2005 now reflects all components of the MyPyramid eating plan, including grains/whole grains, fruit/fruit juice, variety in vegetable and fat types, non/low fat dairy, sodium, and discretionary calories [35]. Traditionally, population HEI-2005 scores have been used with a single 24-hour dietary recall [48], but recently it was shown that it could also be used when multiple recalls were available [49].

The HEI-2005 scores for this population were lower than those shown for individuals 2+ y in 2003-2004 [50]. However, the overall scores of those consuming 100% FJ were significantly higher than those not consuming juice. Consumption of 100% FJ improved total HEI-2005 scores in all age groups; by choosing to consume this single food, the HEI-2005 score improved approximately 10%. However, the total HEI-2005 scores calculated in this study both with and without 100% FJ consumption were still low and needed improvement.

Consumers of 100% FJ also had improved intake of whole fruit. When assessing consumption levels, many studies have combined the intake of fruit and vegetables; however, those studies reporting fruit separately have shown that children, adolescents, or adults [4-6,51] did not meet the fruit recommendation. Data from the 2003-2004 NHANES showed that adult females had the highest percentage (12.3%) of those meeting the fruit recommendation; only 8.6% of adult males met the recommendation [4]. For both genders, the median number of servings consumed was 0.61 per day [4]. Overall consumption was lowest in pre-adolescent, adolescent, and young adult males [5]. Despite extensive, coordinated public health campaigns by government and industry [52], fruit consumption remains low. Consumption of 100% FJ appears to be one way to increase fruit intake; however, the goal should be to encourage intake of all types of fruit.

One concern about consumption of 100% FJ in children and adolescents is that it may replace fluid milk in the diet [53]. Regression analyses showed that there was no difference in the HEI-2005 Milk component score between consumers and non-consumers of 100% FJ. This supports findings from previous studies that consumption of 100% FJ was not associated with lower consumption of milk in the diets of children [15,16].

This study did show that in adolescents 13-18 y consumption of 100% FJ was also associated with higher intake of total, saturated, and discretionary fats. Reasons for this are not clear, but may reflect the overall poor eating habits reported in this age group [43,54]. Additional studies are needed to determine factors leading to poor diet quality in adolescents.

## Limitations

NHANES is a cross-sectional study, thus cause and effect relationships cannot be determined. Participants relied on memory to self-report dietary intakes; therefore, data were subject to non-sampling errors, including under- or over-reporting of energy. Parents reported or assisted their children 2-11 y with the 24-hour recalls; parents often report accurately what children eat in the home [55] but may not know what their children consume outside the home [56], which could also result in reporting errors [57].

## Conclusions

This study showed that, with the exception of children 2-5 y, usual intake of 100% FJ was within the MyPyramid recommendations for children, adolescents, and adults, and the AAP recommendations for children and adolescents. Among consumers, all age groups exceeded MyPyramid recommendations for 100% FJ consumption. Consumption of any amount of 100% FJ was associated with improved diet quality in all age groups. Due to its contribution to overall diet quality, 100% FJ should be recommended to all age groups as a component of a healthy diet.

## Abbreviations

AAP: American Academy of Pediatrics; FNDDS: Food and Nutrient Database for Dietary Studies; HEI-2005: Healthy Eating Index - 2005; FJ: fruit juice; DGAC: Dietary Guidelines Advisory Committee; MEC: Mobile Examination Center; NHANES: National Health and Nutrition Examination Survey; SoFAAS: Solid fats, alcoholic beverages, and added sugars; USDA: United States Department of Agriculture; WIC: Women, Infants, and Children.

## Competing interests

The authors declare that they have no competing interests.

## Authors' contributions

CEO, TAN, and VLF designed this study and had full access to all the data and they take responsibility for the integrity of the data and the accuracy of the data analysis. MZ had full access to all data, worked with collating the data, and with data re-analyses. CEO and TAN were the principal authors. All authors read and approved the final manuscript.
